# A Method Based on Temporal Embedding for the Pairwise Alignment of Dynamic Networks

**DOI:** 10.3390/e25040665

**Published:** 2023-04-15

**Authors:** Pietro Cinaglia, Mario Cannataro

**Affiliations:** 1 Department of Health Sciences, Magna Graecia University of Catanzaro, 88100 Catanzaro, Italy; 2 Department of Medical and Surgical Sciences, Data Analytics Research Center, Magna Graecia University of Catanzaro, 88100 Catanzaro, Italy

**Keywords:** dynamic networks, temporal networks, network alignment, embeddings, temporal embedding

## Abstract

In network analysis, real-world systems may be represented via graph models, where nodes and edges represent the set of biological objects (e.g., genes, proteins, molecules) and their interactions, respectively. This representative knowledge-graph model may also consider the dynamics involved in the evolution of the network (i.e., dynamic networks), in addition to a classic static representation (i.e., static networks). Bioinformatics solutions for network analysis allow knowledge extraction from the features related to a single network of interest or by comparing networks of different species. For instance, we may align a network related to a well known species to a more complex one in order to find a match able to support new hypotheses or studies. Therefore, the network alignment is crucial for transferring the knowledge between species, usually from simplest (e.g., rat) to more complex (e.g., human). *Methods:* In this paper, we present *Dynamic Network Alignment based on Temporal Embedding (DANTE)*, a novel method for pairwise alignment of dynamic networks that applies the temporal embedding to investigate the topological similarities between the two input dynamic networks. The main idea of *DANTE* is to consider the evolution of interactions and the changes in network topology. Briefly, the proposed solution builds a similarity matrix by integrating the tensors computed via the embedding process and, subsequently, it aligns the pairs of nodes by performing its own iterative maximization function. *Results:* The performed experiments have reported promising results in terms of precision and accuracy, as well as good robustness as the number of nodes and time points increases. The proposed solution showed an optimal trade-off between sensitivity and specificity on the alignments produced on several noisy versions of the dynamic yeast network, by improving by ∼18.8% (with a maximum of 20.6%) the Area Under the Receiver Operating Characteristic (ROC) Curve (i.e., AUC or AUROC), compared to two well known methods: *DYNAMAGNA++* and *DYNAWAVE*. From the point of view of quality, *DANTE* outperformed these by ∼91% as nodes increase and by ∼75% as the number of time points increases. Furthermore, a ∼23.73% improvement in terms of node correctness was reported with our solution on real dynamic networks.

## 1. Introduction

In real-world systems, interactions between objects (e.g., molecules) are generally represented through networks. In biology, depending on what the nodes and edges represent, we may classify several types of networks, such as Protein Interaction Network (PIN) [1], Gene Regulatory Network [2] and Signaling Network [3]. For instance, a PIN is a mathematical representation of the physical contacts between the proteins; specifically, each node represents a protein and each edge a Protein–Protein Interaction (PPI) [4,5]. In addition, this representative knowledge-graph model may be applied to investigate the dynamics involved in the evolution of the network (i.e., dynamic networks), in addition to a classic static representation (i.e., static networks). Network analysis allows modeling the dynamics of a network, e.g., to discover the associations between genes and diseases [6] or to explore the interactions between biological objects in a time series [7].

In a dynamic network, the interactions evolve over time [8]. This allows studying both topological and biological hypotheses, in order to investigate evolutionary systems [9]. A “static-temporal approach” allows performing the methods for the analysis of static networks, on dynamic networks [10].

Network Alignment (NA) is generally applied to transfer the knowledge of a simpler network to a more complex one. To give an example, a scientist may apply this approach for describing the functions of gene products related to the human species, starting from the simplest gene network extracted from a set of experiments conducted on the mouse species, with the aim of identifying similar functional and molecular characteristics; in this context, we will assume that the topological similarities between the regions of two given networks correspond to biological processes [11].

NA approaches may be categorized as Local NA (LNA) and Global NA (GNA). LNA looks for small local regions of high similarity (i.e., small subnetworks), admitting a many-to-many node mapping. GNA looks for the best overlapping between the source and target network by aligning large suboptimal subnetworks via a one-to-one (or injective) mapping [12].

Nearly all methods for the alignment of dynamic networks assume that the evolution rate is the same both for the target network and the source network [13]. The alignment of dynamic networks is based on the same notions adopted for the static ones, i.e. exploit an objective function and an optimization strategy to maximize the matching score [14].

According to Thompson et al. [15], a dynamic network can be defined in terms of a multilayer network that includes several dimensions of information on the edges. In this context, an essential one is the time, but others could easily be considered, such as the type of tissue or interaction, to remain in the field of biological networks. Furthermore, the multilayer networks without temporal information are also modeled in many fields, but do not pertain to dynamic networks, nor the context under discussion. However, the dynamic networks make it possible to model continuous data that evolve over time and for which it is possible to study in detail the changes in the network, e.g., referring to stimuli administered/occurring in precise moments or the reactions and times within which these occur and how they impact the network.

In this paper, we present *Dynamic Network Alignment based on Temporal Embedding (DANTE)*, a method for the pairwise alignment of dynamic networks. *DANTE* applies an iterative process for maximizing globally the matching score between the pair of nodes. This work extends our own preliminary prototype [16], optimizing both the NA and the topological similarity evaluation performed via temporal embedding. Furthermore, a novel software tool with a user-friendly Command Line Interface (CLI) was released on our own repository (https://github.com/pietrocinaglia/dante, accessed on 21 March 2023).

Briefly, the main contribution of this work concerns the improvement of the alignment between pairs of dynamic networks, in terms of accuracy and node correctness. To address this purpose, we developed, upstream of the alignment, a method for computing the similarities between the nodes of different dynamic networks based on their topology and related evolution over time. *DANTE* computes all node embeddings by relating these from the source network to the target one. For each node, the temporal embedding considers its topological features and evolution over time. This approach has allowed us to improve the alignment performance.

The main steps performed by DANTE are summarized as follows:

- Evaluating the topological features of a dynamic network based on its node embedding processing over time (i.e., temporal embedding);
- Calculating the similarities of two dynamic networks relating their temporal embeddings;
- Aligning the nodes of a source dynamic network to a target one by maximizing the similarity score.

The rest of the paper is organized as follows. Section 3 presents the design and implementation of *DANTE*, as well as the method applied to build the synthetic dynamic networks used during the tests. Section 4 describes a set of comparative tests both on synthetic and real dynamic networks, useful to evaluate the performance of our solution. Section 5 discusses the results, on the basis of the evidence reported with our method, in the tests. Finally, Section 6 concludes the paper.

## 2. Background

NA is generally based on optimization strategies able to maximize an objective function for mapping the nodes of a source network to a target one. The methods for dynamic networks take their cue from the same formalisms applied to the static ones. They compare pairs of networks in terms of homology and/or topology, over time. For instance, *DYNAMAGNA++* [17] is a well known method for the pairwise alignment of dynamic networks based on *MAGNA++* [18], its equivalent for static ones. *DYNAMAGNA++* evaluates the incident edges and the graphlet changes over time, so that a pair of nodes belonging to two different networks are as similar as their Dynamic Graphlet Degree Vectors (*DGDV*s) are similar. *DYNAWAVE* [19] is an extension of *DYNAMAGNA++* based on *WAVE* [20], another existing method for static networks. Unlike its predecessor, it uses a greedy seed-and-extend strategy for maximizing the Edge Conservation (EC), while the Node Conservation (NC) is handled by applying *DGDV*, as in *DYNAMAGNA++*.

Therefore, both of these solutions produce similar results in terms of NC and are non-deterministic; the latter results in variable performance based on different executions.

Limited to a purely biological context, Time Wrapping algorithm for Aligning Dynamic Networks (Twadn) [21] allows aligning pair of PPI networks. It computes the sequence similarity score between the nodes by using the e-value and bit-score computed via BLASTP [22]. It is therefore important to highlight how this approach works only in the presence of nodes that represent specific biological objects, in this case proteins. Furthermore, the alignment is performed locally and not globally, as in the previous cases.

In recent years, the network embedding was applied for the study of the topological features of both the nodes and the network as a whole [23].

The most relevant model underlying the embedding functioning is the Skip-Gram (SG) model, primarily used in *word2vec* (or Word to Vector) [24] for word embedding. SG is based on a neural network consisting of one hidden layer, for the statistical evaluation of the probability that a word (i.e., target) is present when another one is provided as input. The target word is selected over some rolling window. Therefore, the training data are the pairwise combinations of that target word and all the other words in the window. The result of the SG model yields an embedding for each word or node by analogy.

As an example, *node2vec* (or Node to Vector) [25] applies the SG model to perform the node embedding, by representing each node as a word. Therefore, it extracts a set of random walks from the input graph by representing the walks as a directed sequence of words (i.e., nodes). *node2vec* learns a mapping of nodes to a low-dimensional space of features that maximizes the likelihood of preserving network neighborhoods of nodes.

Note that several implementations of *node2vec* retrieve the embedding vectors by applying directly the model provided by *word2vec*. It works on the same network, by providing information between its own nodes. Similarly, *weg2vec* (or Weighted Event Graph to Vector) [26] allows representing a dynamic network in a reduced dimensional abstract space. It identifies the similarity between the nodes, by predicting both their activation and their influence on a similar set of nodes in the subsequent time point. It works by representing the temporal structure as a static weighted directed acyclic graph, through a Susceptible-Infected (SI) process based on SG.

However, *node2vec* and *weg2vec* cannot be applied to evaluate similarities (or differences) between two or more dynamic networks, but only to relate the objects/nodes within the same network or to embed the features of these into a vector space.

## 3. Materials and Methods

We propose *DANTE*, a method based on temporal embedding that is able to perform the pairwise alignment of dynamic networks. The dynamic networks in input are provided in *snapshot*-based representation, where each snapshot represents a state of the network at a specific time point. Our solution is able to process the similarities between the nodes based on their evolution and relationships over time. For each one, it calculates an embedding vector that represents its features.

It uses the resulting embeddings to model a set of vectors representing the topological similarity between the nodes of two given dynamic networks. Briefly, *DANTE* builds the similarity matrix integrating the tensors computed via the embedding process. Its own alignment function identifies the matches between pairs of nodes by applying the iterative maximization within the similarity matrix.

In the rest of the section, we report the methodology applied to construct the synthetic dynamic networks then exploited in testing. Furthermore, we present the approach at the base of our method for the alignment of pairs of networks based on the topological similarities inferred via temporal embedding.

### 3.1. Construction of Synthetic Dynamic Networks

The construction of the synthetic dynamic networks was based on the Barabási–Albert (BA) model [27]. This is a well known minimal model for scale-free networks, able to generate the related interactions so that each node has at least one link.

We applied it to generate a first time point by defining the following parameters: number of nodes and maximum number of edges for each node.

Subsequently, we generated a snapshot for each time point of interest, based on an evolution rate (*e-rate*). The latter was defined as the randomly shuffling between one time point and the previous one to reproduce a parameterized evolution over time that changes the topology of the network.

At the end of this process, a first dynamic network was correctly built and from this we generated a counterpart network to be aligned.

The second dynamic network was constructed on the basis of the first one in order to form the pair to be aligned.

Depending on the type of test, we kept the same number of nodes or we reduced it by obtaining a sub-structure of the starting network. Regardless of the adopted choice, a noise was introduced to the part of the structure that we kept topologically the same, so that the overall evolution of the network differs by a *delta* value. This approach has allowed us to generate pairs of dynamic networks, of which we can know the best expected alignment on the basis of the generation parameters and therefore evaluate the performance of the alignment tools regarding the desired one.

### 3.2. Temporal Embedding

*DANTE* induces the embeddings based on the SG model. We adapted SG, in order to also support an initial state (i.e., the initial embeddings); for the first time point, the previous state is an empty set. According to the SG model, our approach merges the embeddings belonging to the previous time point in a weight matrix resulting from the intersection of the existing features with the novel one computed on the current time point. For a defined node, the related weighted matrix will reflect the maintenance of the feature over time. The weight matrix is used to process the final embedding that is provided in the output. We describe below how the proposed approach was designed.

We integrated the initial embeddings into the embedding matrix of the SG architecture. Let us denote the initial embeddings via IE and the embedding matrix produced via the SG model based on its input layer via EM, both having the same dimension. The integration (*f*) is performed as the intersection between IE and EM; formally, we merged the embeddings on their weights, taking into account the existing nodes into the initial embeddings, so that the non-intersecting nodes are discarded. Subsequently, the SG model will produce the merged vectors starting from this intersection.

**Figure 1:**
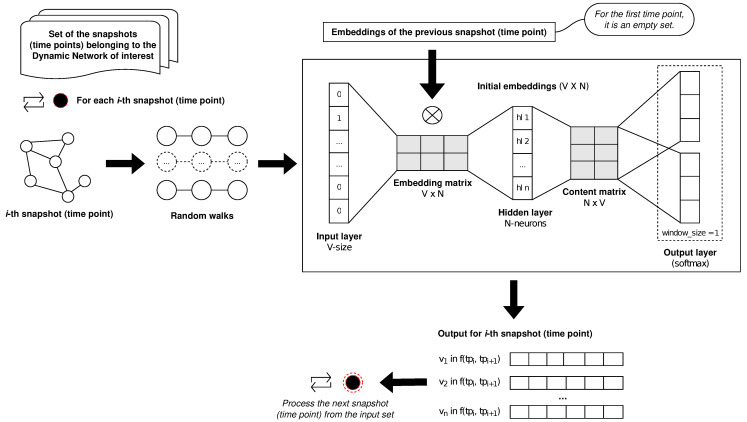
This figure shows how *DANTE* induces the temporal embedding for a dynamic network consisting of *n* time points. Each time point is iteratively processed, by weighting the result based on the previous one, in order to produce a final output that considers the entire temporal evolution. Therefore, this approach allows modeling an outcome that takes into account each state of the dynamic network, over time. In the figure, *f* represents the integration between the embeddings computed for the previous time point and the current one. The modified version of the Skip-Gram architecture is also reported. The latter was extended to support a set of initial embeddings, on the basis of which to produce the current one.

Briefly, each time point is affected by the previous state, which is represented by the output produced by the computation on the previous time point in order to take into account the dynamics of the network. Figure 1 shows how *DANTE* induces the temporal embedding for a dynamic network consisting of *n* time points; *f* represents the integration between the previous time point and the current one, while the first time point is computed by using the original SG model (there is no previous one).

To better explain this step, we introduce the SG model as follows. It consists of a neural network with one hidden layer; there is no activation function. For a set of size *V*, each node in the set is described though a vector. It reports a value equal to one, only for the node of interest.

Let us denote the number of neurons of the hidden layer by *N*; the embedding is a matrix with a dimension of V×N obtained by the dot product between the input and the hidden layer. Furthermore, the output layer performs the dot product between each vector in the embedding matrix (*V* vectors) and its output vector (size equal to *N*), by producing a weighted matrix with size N×V. For each iteration, the model selects a target node over a rolling window (*w*), of which the size represents the context location (*c*) at which the node is predicted. To give an example, the model will evaluate the nodes at c−1 and c+1, for w=1. The SG model uses as activation the *softmax* function (or the *normalized exponential function*) [28] which allows normalizing the output based on a probability distribution [29].

### 3.3. Network Alignment

Let us denote the given dynamic network by G(V,E1), with $V=v_{1},v_{2},\ldots ,v_{n}$ (*n* is the number of nodes in *G*) and the target dynamic network by H(U,E2), with $U=u_{1},u_{2},\ldots ,u_{m}$ (*m* is the number of nodes in *H*); we assumed without loss of generality |V|≤|U|. E1 and E2 are the events (i.e., temporal edges) of *G* and *H*, respectively.

The pairwise alignment of *G* and *H* is performed with *DANTE* based on similarities evaluated between nodes of two dynamic networks. Foremost, it evaluates the topological feature of a given node by embedding this one into a vector, which is useful to define its role and position in the network. Therefore, the vector will represent the node and its features. As discussed, the temporal embedding is induced by applying the SG model on each time point, iteratively.

Subsequently, *DANTE* computes the cosine similarity between a simple mean of the projection weight vectors of the given node in *G* and the vectors for each node in *H*. The output consists of a set of vectors, one for each node of *G*, where each vector contains the similarities between the given node of *G* and each node of *H*, sorted in descending order. The latter may also be modeled as a traditional similarity matrix.

*DANTE* performs a one-to-one node mapping (*f*) between *G* and *H*, by maximizing the following objective function (ϕ), applied on each pair of nodes. Therefore, it aligns a node of *G* with a node of *H*, such that no node of *G* maps more than one node of *H* and vice versa. The alignment will provide a set of aligned nodes based on the best match among all vectors, by handling the collisions via the global maximization of *f*.

The mapping f:V→U was implemented by adopting an iterative process, so that it produces a set of aligned node pairs (v,f(v)), with v∈V.

Formally, *f* is computed as follows:

(1) $$f\left(v\right):=\{u|\underset{u\in U}{argmax}\;\phi \left(v,u\right)\}$$

(2) $$\phi \leftarrow cosine\_similarity\left(v,u\right):\{∄\phi \left(v^{\prime },f\left(v^{\prime }\right)\right)\geq \phi \left(v,u\right),v^{\prime }\in V\}$$

### 3.4. Implementation

In this section, we report a brief overview of the implementation of our solution. *DANTE* was implemented in Python3 by using some third-party libraries applied to solve specific issues. We applied the methodology proposed by *node2vec* (see Section 2) for processing the random walks, by extending the SG model to support previous states in the iterative process.

Furthermore, we handled the structure and the dynamic of the network by using *NetworkX* [30], a well known Python package. Similarly, the embedding process is computed by using *Gensim* [31], which allows the training of vector embeddings, while the scientific computing (e.g., cosine similarity) was performed through *Scipy* [32].

### 3.5. Evaluation Criteria

Measures for network alignment are crucial for evaluating the similarity among mapped nodes and the number of conserved edges. According to Chen et al. [10], no accepted criteria to perform an overall comparison between algorithms for NA or motif discovery (both for static and dynamic networks) is generally accepted in the literature; thus, it is hard to say which one is better.

Therefore, we based our considerations on the following set of well known criteria [33]: Node Correctness (NC) and the Area Under the Receiver Operating Characteristic (ROC) Curve (i.e., AUC or AUROC) to evaluate the accuracy of the network alignment. Edge Correctness (EC) is a well known score for static networks, but it is not indicative for dynamic networks; indeed, it can be misleading [19]; therefore, we have excluded it.

## 4. Results

We conducted tests to evaluate the performance of *DANTE*, in comparison with *DYNAMAGNA++* and *DYNAWAVE*. As reported in Vijayan et al. [19], these two well known methods produced similar results in terms of NC for the yeast dataset, due to the use of the *DGDV* method for NC maximization (see Section 2). Therefore, we aggregated their results when evaluating NC, by referring to them by the name of the method they use (i.e., *DGDV*). In contrast, we reported the results separately when evaluating EC, as the performances were different.

In our test, *DYNAMAGNA++* and *DYNAWAVE* have been configured in accordance with their own official documentation (*DYNAMAGNA++, DynaWAVE*, https://www3.nd.edu/~cone/, accessed on 21 March 2023).

We marked as *True Positives* (TPs) the nodes of *G* matched with the namesakes in *H* and *False Positives* (FPs) the node of *G* that did not match with the namesakes in *H*. True Negative (TN) and False Negative (FN) were evaluated based on the basis of the possible matches deriving from the Cartesian product between the nodes of the two networks, for failed matches and truly missing matches, respectively. Note that we repeated each test 10 times by evaluating the average result, for each one.

### 4.1. Test 1—Alignment of Synthetic Dynamic Networks

In the first test, we evaluated the performance of *DANTE* aligning pairs of dynamic networks with different numbers of nodes. Specifically, we constructed each pair of dynamic networks by using the generator described in Section 3.1 and defining the following parameters:

- *number of events for each node:* 2;
- *e-rate:* 0.05 (5%);
- *delta:* 0.10 (10%).

According to our configuration, we may consider the following statements: (i) the topologies between successive time points are ∼95% similar; (ii) in each pair, the dynamic networks are overall ∼90% similar.

Let us denote the source dynamic network by *G* and the target dynamic network by *H*. Referring to the average difference between the complex of the time point in *G* and the complex of the ones in *H*, the difference between two time points is the *delta* parameter defined in our generator and reported above. As described in Section 3.1, the topology of *H* is partly preserved in *G*. Briefly, the latter is constructed starting from the former, by introducing a low noise. According to the defined parameters, we will expect that the alignment will match most of the nodes in *G* to their own namesakes in *H* (e.g., $v_{1}$ in *G* to $v_{1}$ in *H*, $v_{2}$ in *G* to $v_{2}$ in *H* and so on).

Briefly, we constructed the following datasets:

- *Dataset 1*: Three types of pairs (*H*, *G*) consisting of (i) 20 and 10 nodes (i.e., *Pair1*), (ii) 50 and 25 nodes (i.e., *Pair2*), (iii) 100 and 50 nodes (i.e., *Pair3*), for *H* and *G*, respectively. All dynamic networks have 10 time points.
- *Dataset 2*: Four types of pairs (*H*, *G*) having 5, 10, 15 and 20 time points. The dynamic networks all have 100 nodes and the same number of time points within each pair.

We used *Dataset 1* and *Dataset 2* to evaluate the alignment performance as the number of nodes and time points increases, respectively. Each test has been repeated 10 times, by reporting the average result.

**Figure 2:**
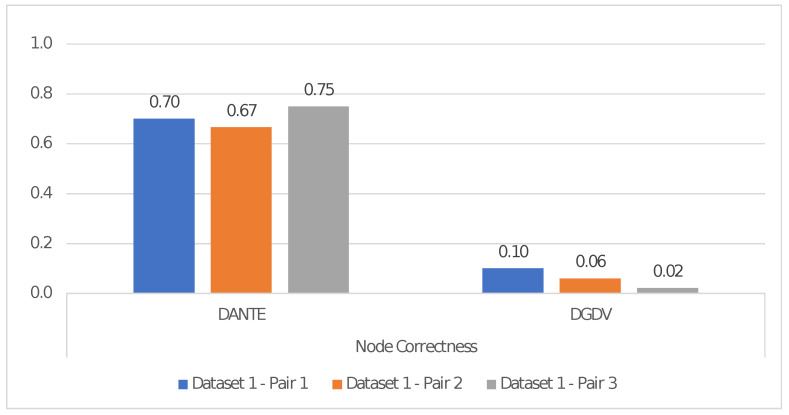
The figure shows NC obtained via *DANTE* and the mentioned *DGDV*-based methods, as the number of nodes increases as 20, 50 and 100, from using *Dataset 1*.

**Figure 3:**
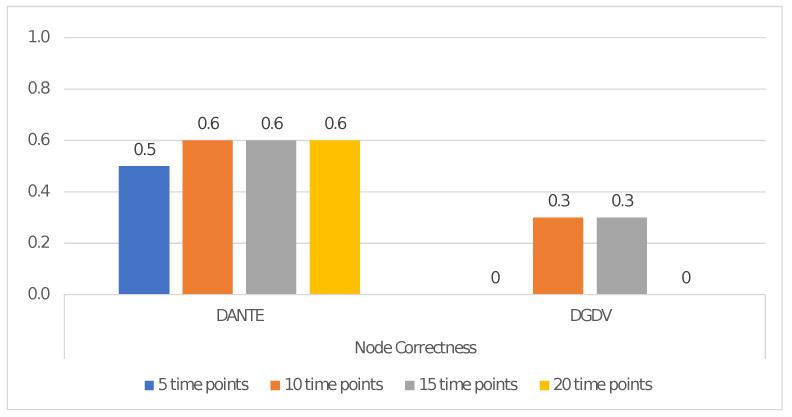
The figure shows NC obtained via *DANTE* and the mentioned *DGDV*-based methods, as the number of time points increases as 5, 10, 15 and 20, from using *Dataset 2*.

We evaluated NC obtained via *DANTE* and *DGDV* (i.e., *DYNAMAGNA++* and *DYNAWAVE*) by taking into account the correctly aligned pairs of nodes (i.e., TP) in all experiments. Figure 2 and Figure 3 show the alignment performance in terms of NC, both as number of nodes and time points increases. These show that *DANTE* outperforms the mentioned *DGDV*-based methods.

### 4.2. Test 2—Alignment of Real Dynamic Networks

In this test, we built a set of 10 real dynamic networks consisting of 100 nodes and 2000 edges spread over eight time points (∼200 events). We randomly extracted data from the real yeast network consisting of 1004 nodes (i.e., proteins) and 8323 interactions spread over eight time points described in [17].

Each resulting dynamic network was aligned with a version of itself containing 0%, 5%, 10%, 15%, 20%, 25% and 30% noise, induced via the random removal of edges and events. Each test was repeated 10 times by reporting the average result.

We performed two main experiments (i.e., *Exp1* and *Exp2*) in order to measure the alignment performance in terms of NC. In *Exp1*, we introduced a specific percentage of noise via the removal of edges within randomly chosen events; in contrast, in *Exp2*, we introduced a specific percentage of noise via the random removal of events. Therefore, in *Exp1*, the interactions remained, having only been degraded over random time points. In *Exp2*, the removal of a random event implied that its interactions over time points were also removed; therefore, the noise is more incisive in this case.

**Figure 4:**
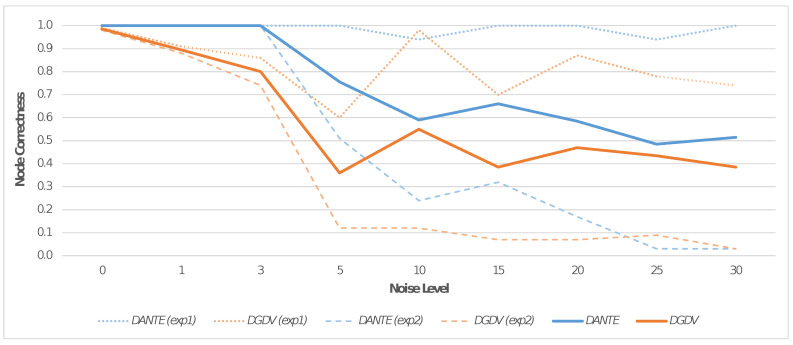
The figure shows a comparison in terms of NC, as the noise increases, both for *DANTE* and *DGDV*. We aligned each proposed dynamic network on a version of itself containing 0%, 5%, 10%, 15%, 20%, 25% and 30% noise, induced via the random removal of edges and events. In the figure, the trend in the foreground is constructed on the basis of the mean between the two experiments (see filled lines).

**Figure 5:**
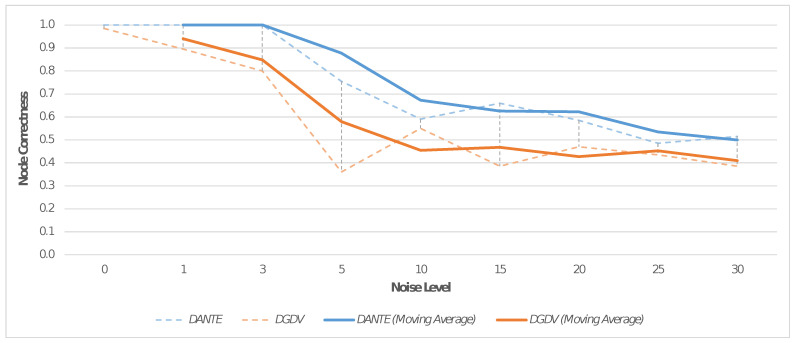
The average trends in Figure 4 were also statistically analyzed, both for *DANTE* and *DGDV*. The resulting trends (see filled lines) were constructed by taking into account the moving average computed for each one. This is symptomatic of the general result of the tests carried out and can be more effectively applied in the comparative context under consideration.

Figure 4 shows a comparison of NC for the two experiments, both for *DANTE* and *DGDV* (i.e., *DYNAMAGNA++* and *DYNAWAVE*). Data were analyzed to determine the NC trend through the statistical study of the moving average, in order to produce an average case that could effectively compare the alignment performance of any other cases. The latter is shown in Figure 5: the resulting trends were constructed by taking into account the moving average computed for each one. This is symptomatic of the general result of the tests carried out and can be more effectively applied in the comparative context under consideration. In addition, the average results for both experiments are reported in Table 1.

**Figure 6:**
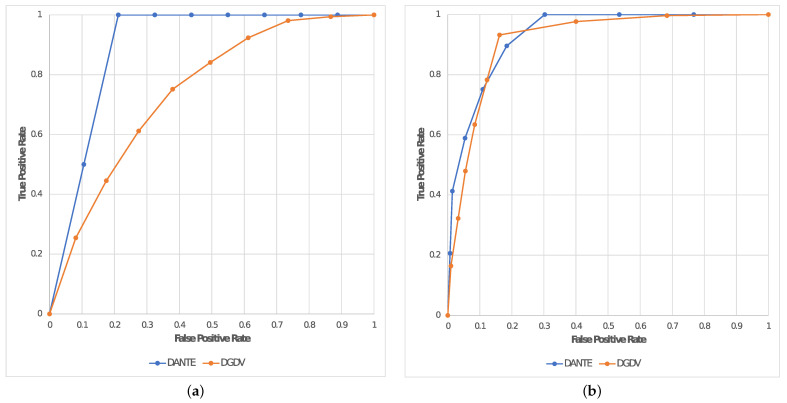

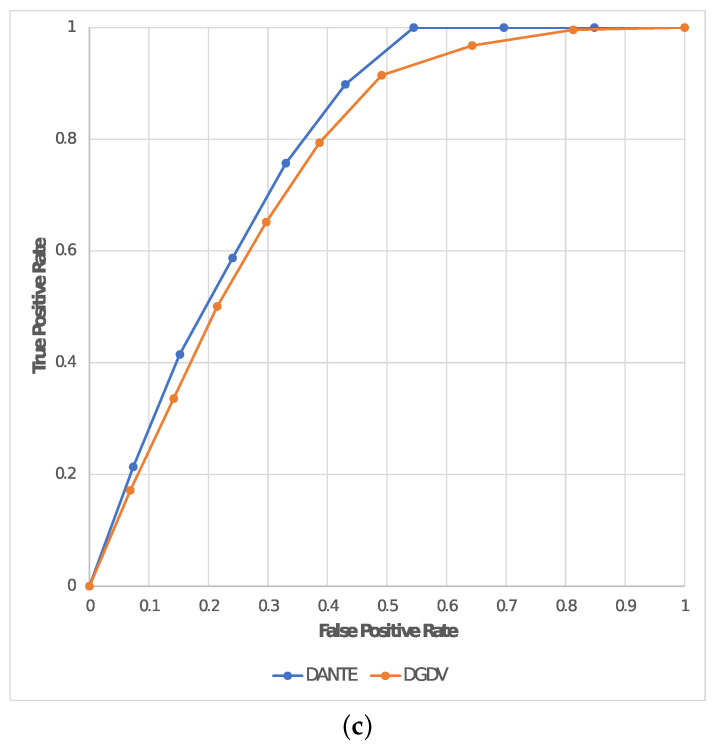
We plotted TPR (or Sensitivity) and FPR (or 1-Specificity) on a dedicated ROC curve for each experiment of *Test 2*. (**a**) A dynamic network is aligned with a noisy version of itself at 0%, 5%, 10%, 15%, 20%, 25% and 30%, induced via the random removal of events. (**b**) A dynamic network is aligned with a noisy version of itself at 0%, 5%, 10%, 15%, 20%, 25% and 30%, induced via the random removal of edges over random time points. (**c**) The curve is built on the average of the results obtained among all the experiments carried out in *Test 2*, in order to provide an overview.

In addition, we evaluated the quality of the alignments in terms of TPR (or Sensitivity) and FPR (or 1-Specificity) in order to also calculate the AUC for each experiment. This is reported in Table 2 by also including the average result between the two experiments; these are plotted in Figure 6 by representing TPR (or Sensitivity) and FPR (or 1-Specificity) on a dedicated ROC curve for each experiment of this test (*Test 2*).

## 5. Discussion

*DANTE* outperformed *DGDV* (i.e., *DYNAMAGNA++* and *DYNAWAVE*), both on synthetic and real dynamic networks.

Regarding the former, it improved the quality of the alignment, in terms of NC, by ∼91% as nodes increase and by ∼75% as the number of time points increases, according to the results shown in Figure 2 and Figure 3. On real dynamic networks, *DANTE* outperformed *DGDV* by ∼23.73% in terms of NC on real dynamic networks (see Table 1). Moreover, it produced an alignment that was on average ∼18.8% better in terms of AUC than the two mentioned solutions based on *DGDV*, reporting a maximum of 20.6% in *Exp1* (see Table 2).

This shows that our solution has proven itself very well, reporting an optimal trade-off between sensitivity and specificity. The AUC resulting from the experiments conducted in *Test 2* (see Table 2) shows the performance metrics in terms of node classification for our solution and *DGDV*. According to Nahm et al. [34], the interpretation of the AUC may be considered as follows:

- AUC≥0.9: Excellent;
- 0.8≤AUC<0.9: Good;
- 0.7≤AUC<0.8: Fair;
- 0.6≤AUC<0.7: Poor;
- 0.5≤AUC<0.6: Fail;
- AUC<0.5: Incorrect.

In *Test 2* we evaluated the performance in terms of NC by calculating the AUC as a qualitative measure. Given the above premises, *DANTE* reported *Good* performance in the first experiment and *Fair* in the second one; due to the fact that the latter has a significantly higher noise level. In contrast, *DGDV* reported *Poor* performance in all experiments, by proving to not be able to report optimal alignments between large dynamic networks.

As discussed in Section 2, *DYNAMAGNA++* and *DYNAWAVE* are non-deterministic; in fact, the tests have shown that this approach degrades the final result on the average of those reported. In contrast to the mentioned solutions, *DANTE* demonstrated better performance for many temporal events, while the former reported better results as the dynamism of the network slows down, tending to a static case. This issue could be due to the fact that the core of both has been built on existing methods for the alignment of static networks (see Section 2).

Ultimately, *DANTE* showed a good robustness as the number of nodes, noise level or time points increased, in terms of precision and accuracy measured in reference to the NC.

## 6. Conclusions

NA allows evaluating different networks in terms of homology and/or topology. It is a method to map nodes from different networks in order to match the same entities. In this paper, we presented a method based on temporal embedding for the pairwise alignment of dynamic networks. We designed and implemented a method able to evaluate the temporal embedding of dynamic networks in order to represent the topological similarities between the nodes. Our solution induces the embeddings via its own customized version of the SG model which supports the modeling of the embeddings on the basis of the evolution of the network over time.

We tested our solution in several use cases based on both synthetic and real dynamic networks, in order to evaluate its performance, in comparison with two well known methods for the alignment of pairs of dynamic networks (i.e., *DYNAMAGNA++* and *DYNAWAVE*). The results were decidedly promising in terms of precision and accuracy, even as the number of nodes and time points increased.

From a point of view of the quality evaluated via NC on synthetic dynamic networks, *DANTE* outperformed *DYNAMAGNA++* and *DYNAWAVE* by ∼91% as nodes increased and by ∼75% as the number of time points increased. On real dynamic networks, *DANTE* outperformed *DGDV* by ∼23.73% in terms of NC. Furthermore, it showed an optimal trade-off between sensitivity and specificity by producing an alignment that was on average better than ∼18.8% in terms of AUC (with a maximum of 20.6%), compared to the two mentioned solutions based on *DGDV*.

## Figures and Tables

**Table 1:** The average values of NC related to the alignment of a dynamic network with a noisy version of itself (*Test 2*). The following levels of noise were applied to the original yeast network: 0%, 5%, 10%, 15%, 20%, 25% and 30%, induced via the random removal of edges and events. In addition, to generate a well formatted plot of the data (see Figure 5), a test on noise yeast network at 1% and 3% was also performed and included.

Noise Level	DANTE	DGDV
0	1	0.99
1	1	0.90
3	1	0.80
5	0.76	0.36
10	0.59	0.55
15	0.66	0.39
20	0.59	0.47
25	0.49	0.44
30	0.52	0.38
*Avg*	*0.73*	*0.59*

**Table 2:** The table reports the average value of AUC for each experiment of *Test 2* by also including the average result between these (i.e., avg). The following levels of noise were applied to the original yeast network: 0%, 5%, 10%, 15%, 20%, 25% and 30%. These were induced via the random removal of edges (i.e., *Exp1*) and events (i.e., *Exp2*).

	Exp1	Exp2	*Avg*
**DANTE**	0.83	0.72	*0.76*
**DGDV**	0.66	0.62	*0.64*
*Improvement*	*+20.6%*	*+16.0%*	*+18.8%*

## Data Availability

*DANTE* is freely available on https://github.com/pietrocinaglia/dante (accessed on 21 March 2023).
